# Identification and Quantification of Anthocyanins in Various Organs of Potato Varieties (*Solanum tuberosum* L.) as Potential Visual Selection Markers During Breeding

**DOI:** 10.3390/plants14132042

**Published:** 2025-07-03

**Authors:** Irina V. Kim, Muhammad A. Nawaz, Dmitry I. Volkov, Aleksey G. Klykov, Mayya P. Razgonova, Kirill S. Golokhvast

**Affiliations:** 1 Federal Scientific Center of Agricultural Biotechnology of the Far East Named after A.K. Chaiki, 30 Volozhenina st., Timiryazevsky stl., 692539 Ussuriysk, Russia; volkov_dima@inbox.ru (D.I.V.); alex.klykov@mail.ru (A.G.K.); 2 Advanced Engineering School (Agrobiotek), National Research Tomsk State University, Lenin Ave, 36, 634050 Tomsk, Tomsk Oblast, Russia; amjad_ucauos@yahoo.com (M.A.N.); golokhvast@sfsca.ru (K.S.G.); 3 N.I. Vavilov All-Russian Institute of Plant Genetic Resources, B. Morskaya 42-44, 190000 Saint-Petersburg, Russia; m.razgonova@vir.nw.ru; 4 Institute of Biotechnology, Bioengineering and Food Systems, Advanced Engineering School, Far Eastern Federal University, 10 Ajax Bay, Russky Island, 690922 Vladivostok, Russia

**Keywords:** anthocyanin profiling, HPLC-MS, Morphological traits, potato breeding, pigmented potato, *Solanum tuberosum* L.

## Abstract

Phenolic compounds, which are a large group of plant pigments, are recognized as important antioxidants. The potato (*Solanum tuberosum* L.), particularly the pigmented varieties, could be a source of natural anthocyanins for producing dietary foods. In this study, we analyzed forty potato specimens from our germplasm collection and breeding nurseries using high-performance liquid chromatography (HPLC) and second-order mass spectrometry to identify anthocyanins. We found seven main anthocyanins in potato tubers: delphinidin-3-glucoside, delphinidin-3-rhamnosyl-5-glucoside, petunidin-3-glucoside, malvidin-3-glucoside, cyanidin-3-glucoside, cyanidin-3-rhamnosyl-5-glucoside, and pelargonidin-3-glucoside. Two anthocyanins were found in potato inflorescences: peonidin-3-coumaroyl glucoside and cyanidin-3-coumaroyl glucoside. On average, varieties from the group with red-purple inflorescences contained 187.6 mg/kg of anthocyanins. Genotypes with white corollas had an anthocyanin content below 0.5 mg/kg or between 1.3 and 3.6 mg/kg. Two potato varieties, Vasilek (605.2 mg/kg) and Fioletovyi (501.1 mg/kg), with blue-purple corollas, had the highest total anthocyanin content. Studying the anthocyanin profile of leaves allowed us to identify eleven anthocyanins. The highest anthocyanin content (331.3 mg/kg) was found in varieties with purple or blue-purple tubers, while the lowest content (an average of 15.1 mg/kg) was found in varieties with yellow or cream tubers. Genotypes with purple and blue-purple tuber skin had an average anthocyanin content of 190.7 mg/kg. The group with yellow and cream tubers had an insignificant anthocyanin content (1.2 mg/kg). Varieties from the group with pink tubers had an average anthocyanin content of 43.2 mg/kg. Thus, this study identified diagnostic traits that could be used to assess the morphological characteristics of potato genotypes.

## 1. Introduction

Phenolic compounds from a large group of plant pigments (anthocyanins) are important antioxidants. Anthocyanins belong to the class of flavonoids and are glucosides of flavylium cations—anthocyanidins [1,2]. Anthocyanidins can be rarely found in the free form in nature. They are synthesized in the skin and flesh of potato tubers [3]. Anthocyanins (of Greek origin; related to *anthos*—a flower and *kyanos*—blue) are a large group of water-soluble plant pigments, which are responsible for red, blue, and purple color of fruits, flowers, leaves, and other plant organs. More than six hundred natural anthocyanins have been identified to date [4]. The glucoside part includes mono-, di-, and tri-saccharides.

Anthocyanins in plants are secondary metabolites [5] and perform important functions, such as tolerance to diverse stressors (biotic and abiotic) [6]. The anthocyanins in the vegetative plant organs (leaves and stems) absorb the visible blue-green spectrum of the light and ultraviolet radiation (UV-B) and serve as powerful antioxidants and neutralizers of free radicals protecting the photosynthetic apparatus from overexposure to visible and ultraviolet radiation and photo-oxidative stress [7]. The anthocyanin content in plants has been reported to increase in response to stresses such as drought, salt, light, UV, oxidative stress, low nitrogen, heavy metals, low temperature, and biotic stressors (bacterial, fungi, viruses, and pests), which trigger the increased expression of early biosynthetic genes and late biosynthetic genes. An increase in the anthocyanin content improves tolerance to biotic and abiotic stresses via removing excess reactive oxygen species, protecting chloroplasts from high-energy quanta, regulating the osmotic balance, and forming chelates [8]. These properties allow plants to adapt to unfavorable conditions such as overexposure to ultraviolet radiation, drought, extreme temperatures, soil salinization, phosphorus and nitrogen deficiency, and the toxic effect of herbicides and heavy metal ions [9]. Anthocyanins show antimicrobial activity and facilitate resistance to wet rot. For example, the diseased area on the surface of the cross section of tubers was 28.6% lower on average in potato with purple flesh than in potato with yellow flesh [10]. Phenolic compounds including anthocyanins are rapidly oxidized in the infected plant tissues and subsequently involved in lignification, suberin deposition, etc. [11].

In addition to their roles in plants’ ability to tolerate stressors, anthocyanins are also used in food and pharmacology industries. The food industry utilizes their properties such as solubility in water and acting as food colorants, thus enabling the producers to color food products in different shades of red (an alternative to carmoisine, a cancerogenic azo-colorant). Anthocyanins can be recommended for coloring some types of cheese, wine, alcohol-free drinks, canned vegetables, breakfast cereals (up to 200 mg/kg), jam, fruit jelly, and jelly candies [12,13]. Consequently, they had been used in medicine to treat and prevent some diseases, e.g., type 2 diabetes, chronic inflammation, hypertension, programmed cell death, etc. [14,15].

It is known that flavonoids accumulate mostly in potato tuber skin, which is rich in compounds of pharmacological interest [16]. It might be beneficial to increase the anthocyanin content in the edible part of potato plants—tuber flesh—for it is an important food crop [17]. Pigmented potato can serve as a potential source of natural anthocyanins due to its low cost and widespread popularity [18]. Red and purple potato contains acylated glucosides of pelargonidin, while purple potato contains acylated glucosides of malvidin, petunidin, peonidin, and delphinidin [19,20]. Potato genotypes differ in the content of biologically active compounds, including anthocyanins. Pigmented varieties are characterized by higher antioxidant capacity (by two to three times) and accumulate more anthocyanins compared to non-pigmented genotypes [21,22]. In terms of cooking and processing, some studies have indicated that cooking and long-term storage do not significantly affect the content of anthocyanins [23]. These qualities of anthocyanins inspire breeders to focus on increasing the anthocyanin content in the red and purple flesh and skin of tubers [24]. In this regard, earlier efforts have successfully developed potato varieties with high carotenoid and anthocyanin contents [25]. In the USA, breeding on such varieties has been conducted since 2000. As a result, new varieties have been created with an enhanced content of carotenoids and anthocyanins and red and purple tuber flesh [2]. Generally, red and purple tuber flesh contains anthocyanins such as pelargonidin, malvidin, petunidin, peonidin, and delphinidin [26]. Moreover, the anthocyanin content in potato has been reported to be 5.5–35 mg/100 g of the dry matter [13,27]. Russian breeders have achieved significant success in creating potato varieties for dietary nutrition as well; research has been carried out to determine the total content of anthocyanins in potato specimens. Some varieties with the highest index of pigmentation were discovered to contain five times more antioxidants than varieties with white flesh [28]. A positive correlation has been established between the content of anthocyanins and other phenolic compounds in the tissues of potato tubers and the antioxidant activity [29]. The Federal Scientific Center of Agricultural Biotechnology of the Far East named after A.K. Chaiki, Ussuriysk, Russia, has been carrying out research on the breeding of pigmented potato as well [30].

Effective breeding based on tuber flesh color (red and purple) is associated with the development of DNA markers for the target genes of anthocyanin biosynthesis. Recent research has indicated that both the early biosynthesis and late biosynthesis genes [2,31] and their regulators, i.e., R2R3 MYB transcription factors (e.g., StFlAN2), are the key players in anthocyanin biosynthesis in potato [32,33]. The transfer of StFlAN2 into homozygous monoploid plants with white flowers has restored the homochromous purple pigmentation of the corolla [31,34]. Considerable knowledge on the genetics of the anthocyanin pigmentation in *S. tuberosum* tubers and flowers substantially simplifies the selection of parental pairs for hybridization and the calculation of the needed amount of original material when selecting hybrids with pigmented flowers, stems, leaves, and tubers [35]. The correlation between the color of the corolla and the MYB transcription factors has been described in details for a large number of flowering plants [21,36,37,38,39]. According to Simakov E.A. et al. [40], who has assessed the antioxidant activity (AOA) in a variety specimens from a collection nursery, a high level of AOA (1032–1280 mg/kg) has been found in hybrids with pigmented tuber skin and flesh and a high level of carotenoids and anthocyanins. The hybridization of parental forms with red-purple and red tubers has resulted in a higher number of phenotypes (by 9.7–12.0%) with red-purple pigmentation among the hybrid progeny than in the variants where homochromous specimens were crossed. Thus, studying a diverse set of potato germplasms would allow for the selection of plant material for breeding potato varieties with a high anthocyanin content [20,41].

Nevertheless, the first step in the breeding of potato varieties with a high anthocyanin content and greater antioxidant benefits, and in the development of related molecular markers, would be studying and finding selection traits, e.g., the color of potato plant organs and the type and content of anthocyanins. Here we studied different groups of potato varieties to identify and quantify anthocyanins in different plant organs. These results will provide a theoretical and practical basis for future breeding programs.

## 2. Results and Discussion

### 2.1. Color Variation in Potato Tubers

**Figure 1:**
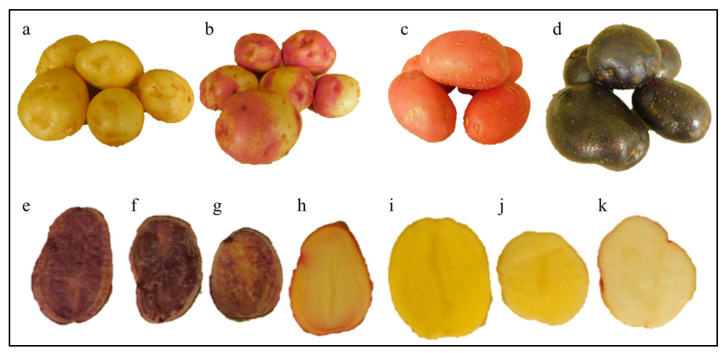
Potato varieties from the groups with a different tuber skin color: (**a**) Sante, st; (**b**) Ol’skii; (**c**) Manifest; and (**d**) Chernyi prints. (**e**,**f**) Differences in the tuber flesh color among the studied potato specimens: Chernyi prints (**e**); Tsyganka Lora (**f**); Vasilek (**g**); Pri-15-12-14 (**h**); Yantar’, st (**i**); Sante, st (**j**); and Ol’skii (**k**).

Morphological traits can be a good criterion for the selection of parents for the efficient breeding of potato to develop hybrids and new varieties with a high anthocyanin content [42]. The first step in our experiment was to evaluate the specimens for the color of the tuber skin and divide them into three groups. The first group included 17 specimens with yellow and cream tuber skin. The second group included 17 specimens with yellow tuber skin with pink spots and pink and red tuber skin. The third group included six specimens with purple and blue-purple tuber skin (Figure 1a–d). The studied genotypes differed in their morphological traits (Table 1). These results suggest that the studied potato varieties are diverse in skin color. Several potato varieties from different countries have been reported to have yellow, red, blue, red-blue (Turkey) [43], and white and red tuber skin (Iran). This phenomenon has also been observed in sweet potato tubers, e.g., varieties from Indonesia have shown yellow, orange, cream, dark-orange, dark-purple, white, and purple-white skin colors [44].

**Figure 2:**
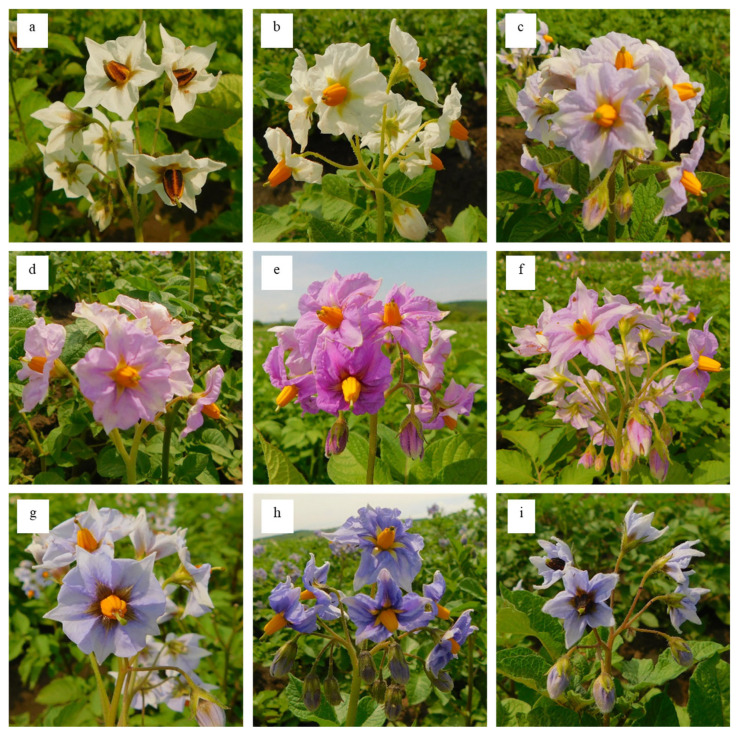
Variation of the corolla color among the studied potato specimens: (**a**) Sarma (white); (**b**) Kazachok, st (white); (**c**) Krepysh (pale red-purple with white streaks); (**d**) Pamyati Rogacheva (pale red-purple); (**e**) Yubilyar (red-purple); (**f**) Pamyati Rogacheva (pale red-purple); (**g**) Pri-12-35-4 (blue-purple); (**h**) Nadezhda (blue-purple with white streaks); (**i**) Chernyi prints (red-purple with white streaks).

Earlier research on tuber flesh has shown that potato tubers differ in their nutritional content and nutraceutical value [45], connection with candidate genes [46], and the presence of carotenoids and anthocyanins [47]. Since tuber flesh is the potato part that is most commonly used for human nutrition, the selection of potato specimens with a specific color would be appropriate for breeding highly nutritious varieties. The varieties examined in this research exhibited different colors, i.e., white, cream, yellow, pink, and purple (Figure 1e–k). Nine of the studied varieties had white tuber flesh, ten had cream flesh, and twenty had a yellow flesh color of different intensities and purple pigmentation. Only one specimen had purple flesh—Fioletovyi (Table 1). However, since tubers are underground parts, a breeder would need non-destructive morphological traits, which can be used for the selection of plants before the harvesting stage. Such approaches have proven useful in several plant species and aid in high-throughput phenotyping, e.g., rice [48] and sorghum [49]. In the case of potato, we studied the color of the corolla in inflorescences as the trait that might be used to identify potato genotypes with a high content of anthocyanins at early stages (the flowering stage, 50–60 days after planting). Our results show that the studied varieties had the widest variation—from white to red-purple to blue-purple of different hues (Figure 2).

Fourteen of the studied varieties had a white corolla, twenty-one specimens had a red-purple corolla of different hues, and a blue-purple corolla of different hues was found in five specimens. The morphological traits noted in our research (the color of the generative organs and tuber skin at the flowering stage) were consistent with the descriptions from the literature [50]. In our opinion, the color of different potato plant organs might be considered an important diagnostic trait at the initial stage of breeding, and thus it could be used for the targeted breeding of new varieties.

### 2.2. HPLC-MS-Based Anthocyanin Profiles and Content in Potato Varieties

**Figure 3:**
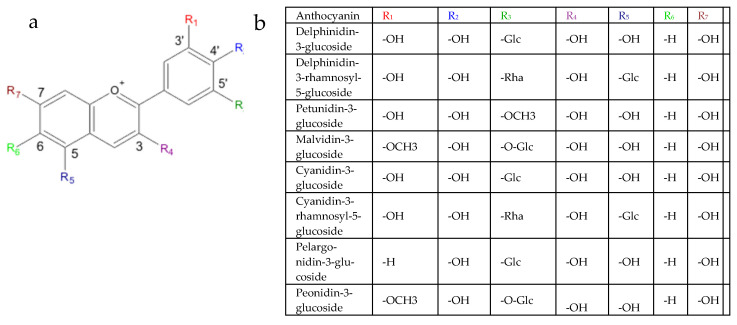
(**a**) Structural formula of anthocyanins (Solominova L.V., Onina S.A., Kozlova G.G. Extraction and study anthocyanins plant material. Bulletin of Science and Practice. 2019. No. 5(4). Pp. 69-75. DOI: 10.33619/2414-2948/41/07). (**b**) Anthocyanins and their respective R groups.

High performance liquid chromatography and its variants, especially HPLC-MS/MS, has become the key method for determining the qualitative and quantitative composition of active compounds [51]. In this study, we employed HPLC-MS to identify anthocyanins and determined their content in potato genotypes of various origin. To this regard, this is the first detailed report on the selected potato varieties. Our results indicate that the studied potato genotypes contained seven main anthocyanins in their tubers: delphinidin-3-glucoside, delphinidin-3-rhamnosyl-5-glucoside, petunidin-3-glucoside, malvidin-3-glucoside, cyanidin-3-glucoside, cyanidin-3-rhamnosyl-5-glucoside, pelargonidin-3-glucoside, and peonidin-3-glucoside. Earlier studies on pigmented potato have also reported the detection of the derivatives of petunidin, pelargonidin, peonidin, and malvidin [52]. The fact that the main anthocyanins detected were glucosides is consistent with studies on other potato varieties. This indicates that glucosides were accumulated in the highest quantities [53] (Figure 3).

#### 2.2.1. Anthocyanin Type and Content in Potato Inflorescences

**Figure 4:**
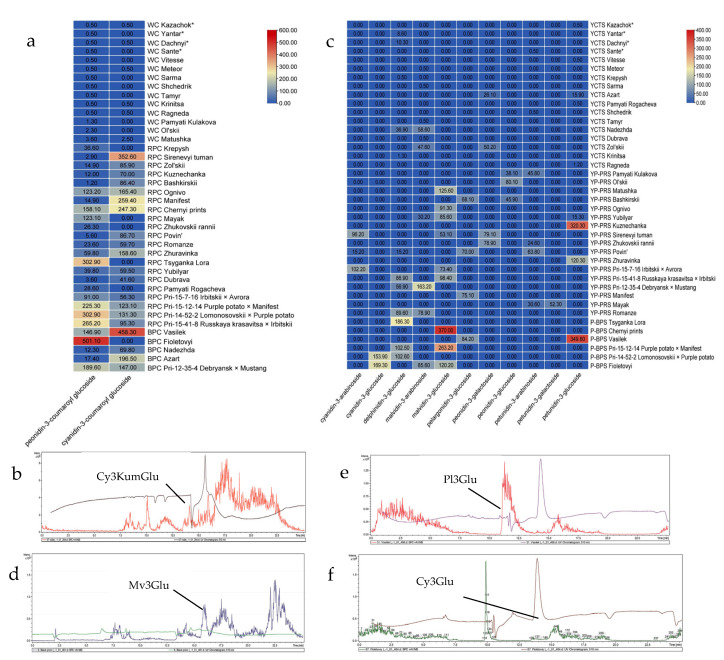
(**a**) Heatmap of the content of anthocyanins in the inflorescences of the studied potato varieties over 2018–2021 (n = 3, *p* < 0.05, M±t0,05½SEM; the wavelength was 510 nm, and this was compared to the spectra of the standards). (**b**) Profiles of the elution of the anthocyanins isolated from the inflorescence of variety Fioletovyi. (**c**) Heatmap of the content of anthocyanins in the leaves of the studied potato varieties. The profiles of the elution of the anthocyanins isolated from the leaves of the varieties (**d**) Chernyi prints, (**e**) Vasilek, and (**f**) Fioletovyi. WC (white corolla), RPC (red-purple corolla), BPC (blue-purple corolla), YCTS (yellow and cream tuber skin), YP-PRS (yellow tuber skin with pink spots, and pink and red tuber skin), and P-BPS (purple and blue-purple tuber skin).

Based on the color of inflorescences, the specimens were divided into three groups: a white corolla, a red-purple corolla (of different hues), and a blue-purple corolla (of different hues). Two anthocyanins were found in the inflorescences: peonidin-3-coumaroyl-glucoside and cyanidin-3-coumaroyl-glucoside. We used four local varieties, i.e., Dachnyi, Kazachok, Yantar’, and Sante, as the standards (Figure 4a).

Figure 4 shows two lines in each chromatogram. These lines indicate that the sample contained two different components, which were successfully separated and detected. As in the case of variety Fioletovyi, the red line with a length of 510 nm in Figure 4b demonstrates the presence of an anthocyanin, i.e., cyaniding 3-coumaroyl-glucoside. The second brown line indicates the presence of an unknown compound in the sample. Depending on the variety, the inflorescences contained one or both compounds. The highest content of anthocyanins (0.3478 g/kg on average) was found in the specimens from the group with a blue-purple corolla, followed by those having a red-purple inflorescence (0.1876 g/kg) and the genotypes with a white corolla (<0.0005 g/kg or within 0.0013–0.0036 g/kg). This is consistent with earlier research indicating that a higher content of peonidin 3-coumaroyl-glucoside imparts red-purple (or deep blue) color [54]. Similarly, a higher content of cyanidin-3-coumaroyl-glucoside in a red-purple inflorescence is consistent with the fact that cyanindin-3-O-glucoisde contributes to red color in plants [55]. Particularly, a relatively higher content of both peonidin-3-coumaroyl-glucoside and cyanidin-3-coumaroyl-glucoside was noted in six specimens, i.e., varieties Vasilek (0.1469 and 0.4583 g/kg); Ognivo (0.1232 and 0.1654 g/kg); Chernyi prints (0.1581 and 0.2473 g/kg); and hybrids Pri-12-35-4 (0.1896 and 0.1470 g/kg), Pri-15-12-14 (0.2253 and 0.1231 g/kg), and Pri-14-52-2 (0.3029 and 0.1313 g/kg). This was consistent with their observed phenotypes (Table 1; Figure 2). Nine varieties, i.e., Azart, Bashkirskii, Zhuravinka, Zol’skii, Manifest, Mayak, Povin’, Sirevyi tuman, and Fioletovyi, contained one anthocyanin component in the inflorescences i.e., cyanidin-3-coumaroyl-glucoside (0.8059–0.5011 g/kg). Two specimens had a higher content of peonidin-3-coumaroyl-glucoside: variety Tsyganka Lora (0.3029 g/kg) and hybrid Pri-15-7-16 (0.910 g/kg). These specimens had light red-purple, red-purple, pale blue-purple, and other colors of the inflorescences. These results indicate that inflorescence color is variable. Other researchers have also reported a wide range of flower colors, i.e., white, light purple, purple, red-purple, purple-red, etc. [56]. The highest total anthocyanin content was found in two varieties with a blue-purple corolla: Vasilek (0.6052 g/kg) and Fioletovyi (0.5011 g/kg) (Figure 4b). The most intensive signal (at a wavelength of 371 nm) (the retention time—25.0 min) corresponded to the main anthocyanin of the variety Fioletoyvi—peonidin-3-coumaroyl-glucoside. Our results provide preliminary data on the anthocyanin content and type in potato inflorescences. The fact that there are limited data on the content and type of anthocyanins in potato flowers makes our results valuable. Such variations in inflorescence color could be due to the differential expression of anthocyanin biosynthesis genes [31]. Further, research should be conducted on the role of genes such as ANTHOCYININ 2 or its homologs in the observed phenotypes. Nevertheless, the early selection of potato varieties based on flower color could be useful as darker colors are associated with higher anthocyanin content (Figure 4).

#### 2.2.2. Anthocyanin Type and Content in Potato Leaves

Potato leaves generate interest for breeders as an additional morphological trait for predicting an increased content of anthocyanins in tubers. This could be used as an early morphological indicator since the vegetative organs of potato develop before the reproductive organs [57]. Since we divided the germplasm from the studied groups based on the color of the tuber skin, i.e., yellow and cream skin (17 specimens), yellow skin with pink spots (17), pink and red skin, purple and blue-purple skin (6), we, therefore, described the results as per this grouping (Table 1). The highest content of anthocyanins in leaves was found in the varieties with purple and blue-purple tuber skin (0.3313 g/kg), and the lowest content (0.0151 g/kg on average) in the specimens from the group with yellow and cream tuber skin. Variety Ol’skii stood out among the other varieties from this group and was characterized by yellow tubers with pink spots and an anthocyanin content of 0.0801 g/kg (Figure 4c). Studies on Chinese potato variety with reddish and purple leaves have reported 520 mg/kg and 680 mg/kg, respectively [21]. However, the differences observed could be due to the varieties studied (genetic background), the growth conditions, and the sensitivity of the quantification methods used [58]. Nevertheless, our results indicate that the anthocyanin content in leaves can depict tuber color such that if the leaves had a higher anthocyanin content, the tubers had a higher pigment content as well (Figure 1 and Figure 4c).

The research on the anthocyanin profile of the leaves identified eleven anthocyanins: petunidin-3-arabinoside, petunidin-3-glucoside, petunidin-3-galactoside, malvidin-3-glucoside, malvidin-3-arabinoside, peonidin-3-galactoside, peonidin-3-glucoside, cyanidin-3-arabinoside, cyanidin-3-glucoside, pelargonidin-3-glucoside, and delphinidin-3-glucoside. These results are somewhat consistent with an earlier work that detected up to 17 anthocyanins in leaves of Chinese potato cultivars [21]. However, the research on the anthocyanin content in potato leaves is rare, and most data are dedicated to sweet potato [59]. Nevertheless, the most common anthocyanin types in the leaves of the studied potato varieties were malvidin-3-arabinoside, malvidin-3-glucoside, and delphinidin-3-glucoside. Our results clearly demonstrate differences between the three groups as well as within the varieties. Some varieties were characterized by complex anthocyanin composition (three-four anthocyanins): Sirenevyi tuman (peonidin-3-galactoside, malvidin-3-glucoside, and cyanidin-3-arabinoside), Fioletovyi (cyanidin-3-glucoside, malvidin-3-glucoside, and malvidin-3-arabinoside), Yubilar (malvidin-3-glucoside, malvidin-3-arabinoside, and petunidin-3-glucoside), and Povin’ (delphinidin-3-glucoside, pelargonidin-3-glucoside, petunidin-3-arabinoside, and cyanidin-3-arabinoside). Among the standard varieties, Yantar’ and Dachnyi were characterized by a very low content of delphinidin-3-glucoside in the leaves (0.0086 and 0.0103 g/kg, respectively). Previous research on other species regarding the variation in the anthocyanin content in leaves has also depicted inter-varietal differences [59].

Genotypes with the highest anthocyanin content included Bashkirskii (0.1140 g/kg); Vasilek (0.4340 g/kg); Zhukovskii rannii (0.1035 g/kg); Zol’skii (0.0978 g/kg); Kuznechanka (0.3203 g/kg); Matushka (0.1256 g/kg); Mayak (0.0829 g/kg); Nadezhda (0.0955 g/kg); Pamyati Kulakova (0.0839 g/kg); Sirenevyi tuman (0.2284 g/kg); Tsyganka Lora (0.1863 g/kg); Fioletovyi (0.3751 g/kg); Chernyi prints (0.3700 g/kg); Yubilar (0.1311 g/kg); Povin’ (0.1642 g/kg); Romanze (0.1685 g/kg); and hybrids Pri-12-35-4 (0.2201 g/kg), Pri-15-12-14 (0.3657 g/kg), Pri-14-52-2 (0.2565 g/kg), Pri-15-7-16 (0.1756 g/kg), and Pri-15-41-8 (0.1853 g/kg). These varieties had pigmented tubers and skin—pink, red, purple, and blue-purple (Figure 4d–f). The most intensive signal (at a wavelength of 510 nm) (the retention time—25.0 min) corresponded to the main anthocyanin of variety Chernyi prints—malvidin-3-glucoside (peak at 14.6 min); variety Vasilek—pelargonidin-3-glucoside (peak at 14.1 min) and petunidin-3-glucoside (peak at 18.2 min); and variety Fioletovyi—cyanidin-3-glucoside (peak at 13.4 min), malvidin-3-glucoside (peak at 18.5 min), and malvidin-3-arabinoside (peak at 23.5 min). Their presence corresponds to respective colors of these anthocyanins in plant tissues [60,61]. Since the genotypes with the pigmented tubers of pink, red, and purple hues had an enhanced content of anthocyanins in the leaves, it could be suggested that leaf color (and the anthocyanin content) is a trait of interest for the early selection of potato varieties with a high anthocyanin content in tubers.

#### 2.2.3. Anthocyanin Type and Content in Potato Tubers

**Figure 5:**
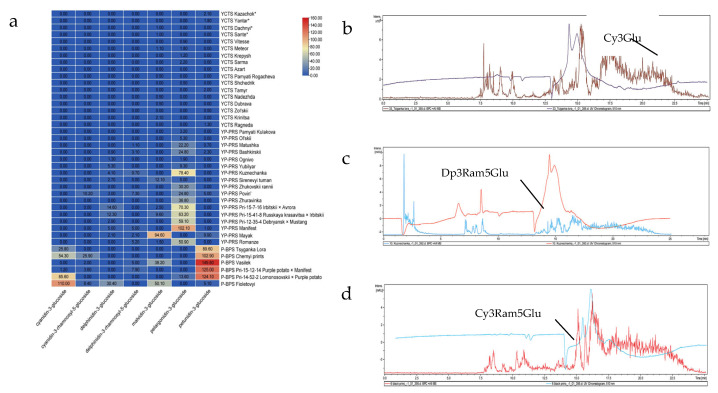
(**a**) Content of anthocyanins in the tubers of the studied potato varieties depending on skin color over 2018–2021 (n = 3, *p* < 0.05, M ± t0.05½SEM; the wavelength was 510 nm, and this was compared to the spectra of the standards). The profiles of the elution of the anthocyanins isolated from the tubers of the studied potato varieties (**b**) sort Tsyganka Lora, (**c**) Kuznechanka, and (**d**) Chernyi prints. YCTS (yellow and cream tuber skin), YP-PRS (yellow tuber skin with pink spots, and pink and red tuber skin), and P-BPS (purple and blue-purple tuber skin).

Wang H. et al. [62] has established that petunidin and pelargonidin are the main anthocyanins in potato tubers. Petunidin is responsible for purple pigmentation and pelargonidin for orange-red pigmentation [61]. At the initial stage of the experiment, we analyzed unpeeled potato tubers (the content of anthocyanins in tuber flesh and skin as a whole). Our results demonstrate that the varieties with pink and dark pink tubers had higher pelargonidin-3-glucoside content, while the varieties with purple and blue-purple tubers contained mainly petunidin-3-glucoside and cyanidin-3-glucodise. A nonsignificant quantity of anthocyanins was detected in the specimens with yellow tuber skin (Figure 5a).

The chromatogram in Figure 5b shows the presence of two compounds, which are represented by lines of different colors. The dark red line with a length of 510 nm demonstrates the presence of an anthocyanin, i.e., cyaniding-3-glucoside, while the brown line indicates the presence of an unknown compound in the sample.

The group with purple and blue-purple skin had the highest average anthocyanin content (0.1907 g/kg), followed by those with the pink pigment in the tubers (0.0432 g/kg) and yellow and cream tubers (0.0012 g/kg). Studies on German potato varieties have shown an anthocyanin content of up to 0.6500 g/kg in skin, 0.3100 g/kg in whole tubers, and 0.2200 g/kg in flesh [63]. However, the differences in our results from those of other studies could be due to several factors such as genotype, environment and growth stage, nutrition and agronomic practices, etc., [64,65].

The most intensive signal (at a wavelength of 510 nm) (the retention time—35.0 min) corresponded to the main anthocyanin of variety Tsyganka Lora—petunidin-3-glucoside. Additionally, cyanidin-3-glucoside was found in considerable quantity (peak at 27.5 min). The main anthocyanins of variety Kuznechanka were delphinidin-3-glucoside (peak at 25.0 min), delphinidin-3-rhamnosyl-5-glucoside (peak at 17.5 min), and pelargonidin-3-glucoside (peak at 37.5 min) and of variety Chernyi Prints were cyanidin-3-glucoside (peak at 27.5 min), cyanidin-3-rhamnosyl-5-glucoside (peak at 19.0 min), and petunidin-3-glucoside (peak at 35.0 min). Moreover, our results indicate that potato varieties also differ in their anthocyanin composition. For example, Bashkirskii was characterized by delphinidin-3-glucoside, delphinidin-3-rhamnosyl-5-glucoside, pelargonidin-3-glucoside, and petunidin-3-glucoside. Vasilek was characterized by delphinidin-3-rhamnosyl-5-glucoside, pelargonidin-3-glucoside, petunidin-3-glucoside, and cyanidin-3-rhamnosyl-5-glucoside. Generally, varieties with skin color ranging from purple to blue-purple were richer in petunidin-3-glucoside, cyanidin-3-glucoside, delphinidin-3-glucoside, and malvidin-3-glucoside. In contrast, those with yellow tuber skin with pink spots as well as pink and red tuber skin were characterized by a higher content of pelargonidin-3-glucoside, malvidin-3-glucoside, delphinidin-3-rhamnosyl-5-glucoside, delphinidin-3-glucoside, and cyanidin-3-rhamnosyl-5-glucoside (Figure 5a). Earlier studies have indicated inter-varietal differences in the anthocyanin content in tubers [52]; we also observed such differences. The highest content of anthocyanins was characteristic of the specimens with pink tuber skin (Kuznechanka—0.0922 g/kg, Pri-15-7-16—0.0874 g/kg, Pri-15-41-8—0.0851 g/kg), red skin (Mayak—0.1087 g/kg), purple skin (Tsyganka Lora—0.1154 g/kg, Chernyi prints—0.1831 g/kg, Vasilek—0.1950 g/kg, Pri-15-12-14—0.1377 g/kg, Pri-14-52-2—0.2233 g/kg), and blue-purple skin (Fioletovyi—0.2040 g/kg) (Figure 5b–d). These results indicate that the studied varieties had a sufficient anthocyanin content. Future studies should focus on the estimation and determination of the effect that different cooking and processing methods produce on the anthocyanin content [65].

Variety Fioletovyi with blue-purple tubers contained four anthocyanins in considerable quantity: cyanidin-3-glucoside (0.1100 g/kg), malvidin (0.0500 g/kg), delphinidin (0.0304 g/kg), and cyanidin-3-rhamnosyl-5-glucoside (0.0084 g/kg). Specimens Mayak, Kuznechanka, Pri-15-7-16, and Pri-15-41-8 had pink and red tubers and were noted for an enhanced content of pelargonidin—0.0946, 0.0784, 0.0703, and 0.0632 g/kg, respectively. Variety Vasilek with purple tuber skin was characterized by the highest content of petunidin-3-glucoside—0.1498 g/kg. Considering the differences between the content of certain anthocyanins in a given variety, it could be concluded that potato varieties have an unequal nutraceutical value. Variety Fioletovyi had a greater number of anthocyanins in the tubers and, therefore, could be recommended as a source of an entire anthocyanin complex. The pink tubers of varieties Mayak and Kuznechanka and hybrids Pri-15-7-16 and Pri-15-41-8 might be used as sources of pelargonidin, which is known to produce antioxidant, antiatherosclerotic, anti-inflammatory [66], antihyperglycemic, and antidiabetic effects. Pelargonidin could be used as a potential antiobesity agent [67]. It has been reported that pelargonidin shows antitumor activity as well [68]. Variety Vasilek is a good source of petunidin. This anthocyanin is a powerful antioxidant and has the potential to eliminate cancer cells and to lower the risks of a cardio attack [69]. For this reason, the nutraceutical value of potato varieties with purple and pink tubers might be essential for the human diet. Varieties Fioletovyi, Mayak, Kuznechanka, and Vasilek and hybrids Pri-15-7-16 and Pri-15-41-8 could be recommended for use in dietary nutrition to prevent various diseases.

The most intensive signal (at a wavelength of 510 nm) (the retention time—27.5 min) corresponded to the main anthocyanin of this variety—cyanidin-3-glucoside. Additionally, malvidin-3-glucoside (peak at 44 min) and delphinidin-3-glucoside (peak at 25 min) were found in considerable quantity. The profiles of the elution revealed a correlation between the content of corresponding anthocyanins and the tuber color as well as with tbelonging to a given variety. Previous studies have confirmed that the qualitative composition of anthocyanins is usually specific and depends on the variety as well as on the growing conditions, which determine the activity of corresponding enzymes facilitating the synthesis of certain components of the anthocyanin complex [52]. The varieties with purple and pink tuber skin of different hues had a higher content of anthocyanins in their tubers compared to the specimens with yellow tubers. Thus, the color of tuber skin (pink, dark pink, blue-purple, and purple) might be used as a visual diagnostic trait in breeding for the creation of new dietary varieties with an increased content of anthocyanins.

Our results put forward several potato varieties with distinct and useful anthocyanin profiles and contents. These specimens are valuable for their utility as functional food products with high antioxidant activity and could be recommended as original planting material for breeding.

#### 2.2.4. Correlation Between the Anthocyanin Content in Potato Leaves and Tubers

The results presented above on the anthocyanin content in leaves suggest a possible correlation with the anthocyanin content in tubers. To this regard, we further validated this proposition by measuring the correlation between the anthocyanin content from two tissues. The results confirmed our observation that there was a positive correlation between the total content of anthocyanins in these plant organs (Table 1). We also observed that the leaves of the plants that had a pigmented midrib on the upper side contained more anthocyanins. Therefore, there were more anthocyanins in the tubers of these varieties as well. The analysis of the table data showed that the potato varieties whose leaves had the anthocyanin pigmentation of the midrib contained more anthocyanins in the leaves and tubers (from <0.0005 to 0.0103 g/kg and <0.0005–0.0032 g/kg, respectively; r = 0.75–0.95). The only exceptions were the varieties without any positive correlation: Azart (the content of anthocyanins in the leaves was 0.0420 g/kg; r = −0.96), Zol’skii (0.0977 g/kg; r = −0.70), and Nadezhda (0.0955 g/kg; r = −0.85). Only slight traces of anthocyanins were found in the tubers of these varieties.

The varieties with light anthocyanin pigmentation of the midrib predominantly had tubers with pink and red pigmentation. Four specimens were noted to have moderate anthocyanin pigmentation of the midrib: Kuznechanka (the content of anthocyanins in the leaves was 0.3203 g/kg, in the tubers—0.0922 g/kg, r = 0.98), Sirenevyi tuman (0.3073 and 0.0198 g/kg, respectively; r = 0.75), Pri-15-12-14 (0.3657 and 0.1377 g/kg, respectively; r = 0.97), and Pri-14-52-2 (0.2565 and 0.2233 g/kg, respectively; r = 0.93).

**Figure 6:**
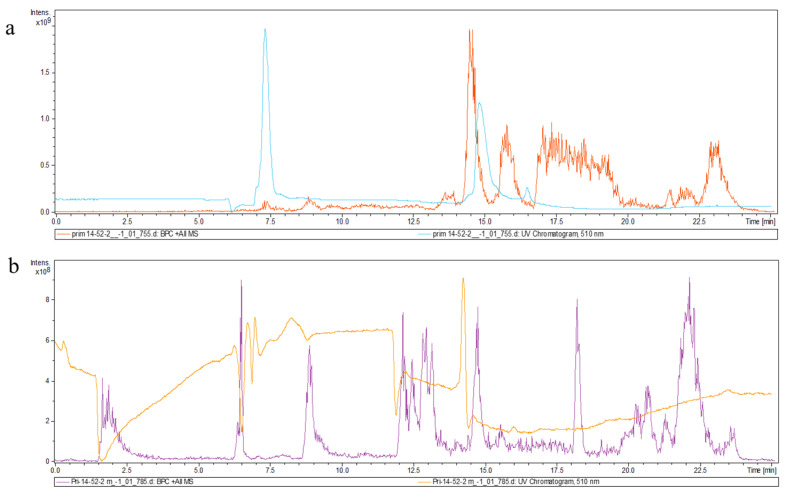
Profile of the elution of the anthocyanins isolated from the tuber skin (**a**) and flesh (**b**) of hybrid Pri-14-52-2.

The varieties with purple and blue-purple tubers were characterized by bright pigmentation of the midrib. The experiment resulted in the identification of four genotypes with similar morphological characteristics: Tsyganka Lora, Chernyi Prints, Vasilek, and Fioletovyi. All four of them had a high content of anthocyanins and a significant correlation between the leaf color and the anthocyanin content in the tubers (r = 0.91–0.97). Thus, the research established a positive correlation between the anthocyanin pigmentation of potato leaves and tubers and the total content of anthocyanins (Figure 6b).

These results on the positive correlation are consistent with an earlier study [70]. Therefore, the anthocyanin content in leaves could prove to be a useful trait for the early selection of potato varieties with a higher anthocyanin content in tubers. Since the anthocyanin content in tubers is also positively correlated with antioxidant activity [71], it can be assumed that those having a higher anthocyanin content in leaves will have higher antioxidant activity as well.

The highest total content of anthocyanins both in the tubers (0.1744 g/kg) and in the leaves (0.3414 g/kg) was characteristic of the group with bright pigmentation of the midrib. This group was characterized by purple and blue-purple pigmentation of tubers. The lowest content of anthocyanins in the both plant organs was noted in the group of the specimens with the leaves that did not have pigmentation (the content of anthocyanins in the leaves on average—0.0499 g/kg; in the tubers—0.0017 g/kg).

Our research results show that the content of anthocyanins was higher in leaves than in tubers in some varieties. A significant difference was noted in the following varieties: Nadezhda, Zol’skii, Matushka, Bashkirskii, Yubilar, Kuznechanka, Sirenevyi tuman, and Pri-12-35-4 Debryansk × Mustang. This might be due to the fact that anthocyanins as pigments often function as protectors against ultraviolet radiation, which predominantly affects upper leaves [2].

The highest correlation coefficient was characteristic of the group with purple and blue-purple tubers (r = 0.94–0.97). The varieties with cream tubers had a low anthocyanin content in the leaves and tubers, which was confirmed by a significant positive correlation (r = 0.86). A low correlation was noted in the varieties with yellow tubers with pink spots (r = 0.39). The specimens with yellow tubers had a moderate correlation (r = 0.52).

The conducted research established a correlation between the anthocyanin pigmentation of the midrib on the upper side of the leaves and an enhanced content of anthocyanins. The pigmentation of leaves might serve as a diagnostic trait for the selection of specimens at the initial stages of potato breeding and at early growth stages (the germination of plants).

#### 2.2.5. Anthocyanin Type and Content in Potato Tubers (Flesh and Skin)

Potato tuber flesh is the plant part that is used for human nutrition. Today the color of tuber flesh ranges from white to purple. Specimens with pigmented tuber flesh are especially valued, for they are sources of antioxidants, including anthocyanins [63]. Two potato varieties with pigmented tuber flesh, i.e., Syurpriz (pink flesh) and Fioletovyi (purple flesh), were included in the State Register of Breeding Achievements (https://gossortrf.ru/registry/, accessed on 12 May 2022) in 2021 and recommended for use in the Russian Federation. However, currently, there are no potato varieties with pigmented tuber flesh that are admitted to use in the Far Eastern region of Russia. This indicates an opportunity for the development of such potato varieties.

When comparing the qualitative and quantitative composition of anthocyanins in the tuber skin and flesh, our results confirmed the hypothesis that the highest content of anthocyanins could be found in tuber skin and in the flesh close to it. The tuber flesh with purple pigmentation had a content of anthocyanins at the same level as the tuber skin (Table 2). These observations are consistent with an earlier report from Germany that the anthocyanin content in skin (0.65 g/kg) was higher that of the whole tubers (0.31 g/kg) or only flesh (0.22 g/kg) [61]. This higher anthocyanin content in peels also corresponded to higher antioxidant activity when compared to flesh [72]. We observed that the genotypes with yellow and cream tuber skin had a nonsignificant average content of anthocyanins—0.0015 g/kg (in the skin) and below 0.0005 g/kg (in the flesh). The coefficient of variation was below 10%, which indicated a low trait variation. The varieties with tuber skin of pink and red hues were characterized by an enhanced anthocyanin content of 0.0738 g/kg in the skin on average and a minimum content of 0.0015 g/kg in the flesh. It was established that the specimens with purple tubers contained anthocyanin compounds in the both tuber organs; the average anthocyanin content was 0.3349 g/kg in the skin and 0.0803 g/kg in the flesh.

Analyzing the composition of the tuber organs resulted in the identification of the following potato specimens containing anthocyanins both in the tuber skin and flesh: Vasilek (petunidin-3-glucoside—0.1498 g/kg in the skin and 0.0261 g/kg in the flesh), Tsyganka Lora (petunidin-3-glucoside—0.0896 g/kg and 0.0126 g/kg, respectively), Fioletovyi (cyanidin-3-glucosode—0.1100 g/kg and 0.0352 g/kg), Chernyi prints (cyanidin-3-glucoside—0.0543 g/kg and 0.0103 g/kg; petunidin-3-glucoside—0.1029 g/kg and 0.0241 g/kg), Pri-15-12-14 (petunidin-3- arabinoside—0.3100 g/kg and 0.0911 g/kg; cyanidin-3-glucoside—0.1498 g/kg and 0.0398 g/kg), and Pri-14-52-2 (petunidin-3-arabinoside—0.3600 and 0.1430 g/kg; cyanidin-3-glucoside: 0.0804 and 0.0812 g/kg). These specimens had pigmented skin.

Flesh with the purple pigment was characteristic of genotypes Fioletovyi, Pri-14-52-2, and Pri-15-12-14. The anthocyanin content found in the specimens with white, yellow, and cream flesh was below 0.0005 g/kg. Hybrids Pri-15-12-14 and Pri-14-52-2 had the highest total content of anthocyanins in the flesh—0.1309 and 0.2242 g/kg, respectively.

In terms of composition, earlier studies have indicated variability within potato of different colors [53]. Anthocyanins from skin and flesh differ in their composition and content [54,72]. We observed similar results. For example, hybrids Pri-15-12-14 and Pri-14-52-2 exhibited the presence of various types of anthocyanins in different quantities. The content of cyanidin-3-glucoside was 0.1498 g/kg and 0.0702 g/kg, respectively, in the skin. Its content in the flesh was 0.0398 g/kg and 0.0812 g/kg, respectively. In contrast, the content of petunidin-3-arabinoside in the skin of the hybrids was 0.31 g/kg and 0.36 g/kg, respectively. Its content in the flesh of the two hybrids was 0.0911 g/kg and 0.1430 g/kg, respectively (Table 2).

Petunidin-3-arabinoside was detected in the tuber skin and flesh of hybrid Pri-14-52-2 in significant quantity and found to be the main component in the tubers of this specimen (Figure 6).

An intensive signal was detected at a wavelength of 510 nm (the retention time—15.5 min) that corresponded to the main anthocyanin of hybrid Pri-14-52-2—petunidin-3-arabinoside. Additionally, pelargonidin-3-glucoside (peak at 14.9 min) was found in significant quantity. When the tuber flesh of hybrid Pri-14-52-2 was analyzed, we detected a peak with an intensive signal at 14.3 min that corresponded to petunidin-3-arabinoside. Another signal was detected at 10.5 min where cyanidin-3-glucoside was identified. The profiles of the elution revealed a correlation between the content of certain anthocyanins and the color of tubers as well as with their belonging to a certain variety.

The literature confirms that the qualitative composition of anthocyanins depends on the characteristics of a given genotype, which determine the activity of corresponding enzymes facilitating the synthesis of certain components of the anthocyanin complex [53].

## 3. Material and Methods

### 3.1. Plant Material

The research was conducted on forty potato specimens from the germplasm collection and breeding nurseries of the Federal Scientific Center of Agricultural Biotechnology of the Far East named after A.K. Chaiki, Ussuriysk, Russia, in 2018–2025. The potato specimens were selected based on their productivity (≥500 g/plant) and the color of tuber skin and flesh (Table 3). The color of the tuber skin and flesh was determined visually after harvesting in laboratory conditions. Anthocyanins were identified according to the method described by Kim et al. [73]. The anthocyanin identification was carried out at the Far Eastern Federal University, Vladivostok, Russia.

### 3.2. Potato Specimen Preparation

To determine the content of anthocyanins, we used tuber skin and flesh, leaves, and inflorescences. The collected specimens were stored in a cool dark place until the analysis was performed. The samples were rinsed in cold water, weighted, diced, and submerged into a solution containing 40% ethanol and 1% formic acid (5 g of the diced mass + 25 mL of the solution). The mass was subjected to freezing-thawing, i.e., frozen at −80 °C in an ultra-low temperature freezer UF V700 (Binder, Germany) and thawed in a box without exposure to light at a temperature of +19–22 °C, followed by ultrasound disintegration to destroy the walls and membranes of cells and organelles. Anthocyanins were extracted in a closed vessel (to prevent exposure to atmospheric oxygen) at 40 °C for 90 min. The extracts were centrifuged (CM-6M, “Elmi”, Latvia) at 3500 g for 30 min. The supernatant was filtrated through membrane filters (pore size—0.45 μm). The obtained extract was stored at −20 °C in a freezer.

### 3.3. Anthocyanin Separation by HPLC

The anthocyanins were separated by using a high-performance liquid chromatograph fitted with a high-pressure gradient pump LC-20AD and a prominence column oven CTO-20A (Shimadzu, Kyoto, Japan). The chromatography was performed with a reversed phase column Shodex C18-4E (250 mm × 4.6 mm) with a sorbent particle size of 5 µm (Shodex, Tokyo, Japan) at a temperature of 50 °C and a flow rate of 0.58 mL/min. Acetonitrile (AppliChem GmbH, Darmstadt, Germany) was used as the eluent A, whereas the eluent B was a 1% formic acid aqueous solution (Sigma-Aldrich, Saint Louis, MO, USA). The gradient program was as follows: 0.00–5.00 min—the concentration of B decreased from 100% to 92%; 5.00–45.00 min—the concentration of B decreased from 92% to 80%; 45.00–45.01 min—the concentration of B decreased from 80% to 10%. The column was washed after each injection. The detection was conducted within a wavelength rage of 300-600 nm employing a UV–VIS detector SPD-20A (Shimadzu, Japan). We injected 5 μL of the extract for the analysis. The content of anthocyanins in varieties Fioletovyi and Vasilek was quantified based on pure malvidin-3-glucoside using a molar attenuation coefficient of 3.02 × 10^4^ and on pure cyanidin-3-glucoside using a molar attenuation coefficient of 2.69 × 10^4^ as the control at a wavelength range of 300–600 nm and a molecular mass of 493.3 g/mol. The content of anthocyanins in the other varieties was quantified based on pure pelargonidin-3-glucoside as the control using a molar attenuation coefficient of 2.73 × 10^4^ at a wavelength range of 300–600 nm and a molecular mass of 433.3 g/mol. All the quantifications were done in triplicate.

### 3.4. Identification of Anthocyanins

The anthocyanins were identified by HPLC-MS employing an ion trap amaZon SL (“Bruker”, Bremen, Germany) equipped with an electrospray ion source. For this purpose, the anthocyanins separated earlier by HPLC were analyzed by the method of direct injection. The detection was conducted in the regime of positive and negative ions. The mass range was from 150 to 2200 *m*/*z*, the highest scan speed was 32,000 u/s, the voltage on the electrospray emitter was 4500 V, the pressure on the nebulizer was 29 psi, the dry gas flow rate was 10 l/s, and the temperature of the emitter was 180 °C. The fragmentation of ions was performed by a 1.5 eV electron beam.

### 3.5. Statistical Analysis and Visualization

Plant sample figures were assembled in Microsoft PowerPoint 2024 Professional Plus (www.microsoft.com, accessed on 3 February 2023). Heatmaps were generated in TBtools (https://bio.tools/tbtools, accessed on 12 May 2022) [42]. To confirm the validity of the research results, we performed one-factor ANOVA and subsequently employed Fisher’s LSD method for multiple comparisons using MS Excel 2007 and Statistica 10 (StatSoft, Inc., Tulsa, OK, USA). Duncan’s multiple range test (*p* < 0.05) was used to measure the significance of differences. The correlation coefficient was calculated according to Pearson [74]. Results were expressed as mean values ± standard deviation. The analysis was performed with 120 biological and 3 analytical repeats (n = 3).

## 4. Conclusions

Our research identified the selection traits that might be used for assessing the morphological characteristics of potato genotypes. The anthocyanin pigmentation of the midrib on the upper side of potato leaves can be considered as a diagnostic trait when selecting potato specimens with an enhanced anthocyanin content at early developmental stages of plants (the germination of the majority of potato plants). The pigmentation of the corolla in the potato inflorescences, especially red-purple and blue-purple pigmentation, indicated the presence of anthocyanins in the potato tubers. This characteristic can be used for the selection of specimens at the flowering stage. It was established that the tubers with pink and dark pink skin were characterized by the presence of pelargonidin-3-glucoside. Petunidin-3-glucoside and cyanidin-3-glucoside were responsible for purple and blue-purple pigmentation of the tuber skin. Detecting the anthocyanin profile in the different organs of potato plants resulted in the identification of potato varieties that might be used as sources of an enhanced anthocyanin content: Vasilek, Kuznechanka, Manifest, Mayak, Tsyganka Lora, Fioletovyi, Chernyi prints, Pri-15-12-14 Purple potato × Manifest, and Pri-14-52-2 Lomonosovskii × Purple potato. The research allowed us to obtain effective combinations and breeding material. The selected potato specimens could be recommended for dietary nutrition and further use in breeding to create varieties with an enhanced content of anthocyanins. These preliminary results provide future opportunities on testing the genotypes to understand and confirm if the anthocyanin content changes during processing and cooking. The extreme phenotypes with white and purple flowers and corresponding tuber skin and flesh colors might be ideal parents for developing populations for genome-wide association studies. Such studies would enable the development of molecular markers for marker-assisted selection and gene characterization.

## Figures and Tables

**Table 1:** Content of anthocyanins in the leaves and tubers of the studied potato varieties over 2018–2021 (n = 3, M ± t0,05½SEM).

Variety/Hybrid	Leaf		Tuber	Correlation Coefficient * (r)
	Anthocyanin Pigmentation of the Midrib on the Upper Side	Total Content of Anthocyanins, g/kg	Total Content of Anthocyanins, g/kg	
Yellow and cream tuber skin				
Kazachok, st	None	<0.0005	0.0021 ± 0.0001	0.85
Yantar’, st	None	0.0086 ± 0.001	0.0018 ± 0.0001	0.78
Dachnyi, st	None	0.0103 ± 0.001	0.0010 ± 0.0001	0.75
Sante, st	None	<0.0005	0.0010 ± 0.0001	0.85
Vitesse	None	<0.0005	0.0009 ± 0.0001	0.80
Meteor	None	<0.0005	0.0029 ± 0.0001	0.85
Krepysh	None	<0.0005	0.0012 ± 0.0001	0.86
Sarma	None	<0.0005	0.0022 ± 0.0001	0.90
Azart	None	0.0420 ± 0.001	<0.0005	−0.96
Pamyati Rogacheva	None	<0.0005	<0.0005	0.92
Shchedrik	None	<0.0005	<0.0005	0.95
Tamyr	None	<0.0005	0.0020 ± 0.0001	0.85
Nadezhda	None	0.0955 ± 0.002	<0.0005	−0.85
Dubrava	None	<0.0005	<0.0005	0.98
Zol’skii	None	0.0977 ± 0.002	<0.0005	−0.70
Krinitsa	None	0.0013 ± 0.001	0.0021 ± 0.0001	0.86
Ragneda	None	0.0012 ± 0.001	0.0013 ± 0.0001	0.87
Yellow tuber skin with pink spots, pink and red tuber skin				
Pamyati Kulakova	Light	0.0839 ± 0.002	0.0032 ± 0.0001	0.32
Ol’skii	Light	0.0801 ± 0.002	0.0053 ± 0.0001	0.45
Matushka	Light	0.1256 ± 0.003	0,0400 ± 0.0002	0.95
Bashkirskii	Light	0.1140 ± 0.002	0.0311 ± 0.0002	0.74
Ognivo	Light	0.0913 ± 0.002	0.0032 ± 0.0001	−0.56
Yubilar	Light	0.1311 ± 0.003	0.0146 ± 0.0001	0.50
Kuznechanka	Moderate	0.3203 ± 0.005	0.0922 ± 0.0003	0.98
Sirenevyi tuman	Moderate	0.3073 ± 0.005	0.0198 ± 0.0001	0.75
Zhukovskii rannii	Light	0.1035 ± 0.002	0.0302 ± 0.0001	0.52
Povin’	Light	0.1642 ± 0.003	0.0504 ± 0.0002	0.84
Zhuravinka	Light	0.1203 ± 0.002	0.0368 ± 0.0001	0.45
Pri-15-7-16 Irbitskii × Avrora	Light	0.1756 ± 0.003	0.0874 ± 0.0003	0.78
Pri-15-41-8 Russkaya krasavitsa × Irbitskii	Light	0.1853 ± 0.003	0.0851 ± 0.0003	0.85
Pri-12-35-4 Debryansk × Mustang	Light	0.2201 ± 0.003	0.0620 ± 0.0002	0.74
Manifest	Light	0.0751 ± 0.002	0.1131 ± 0.0003	0.68
Mayak	Light	0.0829 ± 0.002	0.1087 ± 0.0003	0.95
Romanze	Light	0.1685 ± 0.003	0.0576 ± 0.0002	0.87
Purple and blue-purple tuber skin				
Tsyganka Lora	Bright	0.1863 ± 0.003	0.1154 ± 0.0003	0.95
Chernyi prints	Bright	0.3700 ± 0.005	0.1831 ± 0.0004	0.91
Vasilek	Bright	0.4340 ± 0.005	0.1950 ± 0.0004	0.95
Pri-15-12-14 Purple potato × Manifest	Moderate	0.3657 ± 0.005	0.1377 ± 0.0003	0.97
Pri-14-52-2 Lomonosovskii × Purple potato	Moderate	0.2565 ± 0.004	0.2233 ± 0.0004	0.93
Fioletovyi	Bright	0.3754 ± 0.004	0.2040 ± 0.0004	0.97

*—0–0.3—very light; 0.31–0.5—light; 0.51–0.7—moderate; 0.71–0.9—bright; 0.91–1.0—very bright.

**Table 2:** Content of anthocyanins in the tuber skin and flesh of the studied potato varieties over 2019–2021 (n = 3, M ± t0.05½SEM).

Variety/Hybrid	Anthocyanin	Molecular Ion [M + H]^+^	Emergence Time	Content of Anthocyanins, g/kg	
				Skin	Flesh
Yellow and cream tuber skin					
Kazachok, st	Petunidin-3-glucoside	479.3; 317.2	35.0	0.0015 ± 0.0001	<0.0005
Yantar’, st	Petunidin-3-glucoside	479.3; 317.2	35.0	0.0012 ± 0.0001	<0.0005
Sante, st	Malvidin-3-glucoside	493.3; 331.3	44.0	0.0012 ± 0.0001	<0.0005
Dachnyi, st	Malvidin-3-glucoside	493.3; 331.3	44.0	0.0019 ± 0.0001	<0.0005
Vitesse	Pelargonidin-3-glucoside	433.3; 271.1	37.5	0.0019 ± 0.0001	<0.0005
Meteor	Malvidin-3-glucoside	493.3; 331.3	44.0	0.0009 ± 0.0001	<0.0005
	Pelargonidin-3-glucoside	433.3; 271.1	37.5	0.0023 ± 0.0001	<0.0005
Krepysh	Pelargonidin-3-glucoside	433.3; 271.1	37.5	0.0015 ± 0.0001	<0.0005
Sarma	Pelargonidin-3-glucoside	433.3; 271.1	37.5	0.0002 ± 0.0001	<0.0005
Azart	Malvidin-3-glucoside	493.3; 331.3	44.0	0.0010 ± 0.0001	<0.0005
Pamyati Rogacheva	Malvidin-3-glucoside	493.3; 331.3	44.0	0.0014 ± 0.0001	<0.0005
Shchedrik	Pelargonidin-3-glucoside	433.3; 271.1	37.5	0.0008 ± 0.0001	<0.0005
Tamyr	Petunidin-3-glucoside	479.3; 317.2	35.0	0.0028 ± 0.0001	<0.0005
Nadezhda	Malvidin-3-glucoside	493.3; 331.3	44.0	0.0013 ± 0.0001	<0.0005
Dubrava	Malvidin-3-glucoside	493.3; 331.3	44.0	0.0012 ± 0.0001	<0.0005
Zol’skii	Petunidin-3-glucoside	479.3; 317.2	35.0	0.0009 ± 0.0001	<0.0005
Krinitsa	Malvidin-3-glucoside	493.3; 331.3	44.0	0.0015 ± 0.0001	<0.0005
Ragneda	Petunidin-3-glucoside	479.3; 317.2	35.0	0.0021 ± 0.0001	<0.0005
Mean				0.0015	<0.0005
V, %				9.7	0.1
Yellow tuber skin with pink spots, pink and red skin					
Pamyati Kulakova	Pelargonidin-3-glucoside	433.3; 271.1	37.5	0.0056 ± 0.0001	<0.0005
Ol’skii	Pelargonidin-3-glucoside	433.3; 271.1	37.5	0.0070 ± 0.0001	<0.0005
Matushka	Delphinidin-3-rhamnosyl-5-glucoside	627.3; 465.3; 303.2	17.5	0.0026 ± 0.0001	<0.0005
	Pelargonidin-3-glucoside	433.3; 271.1	37.5	0.0308 ± 0.0002	<0.0005
	Petunidin-3-glucoside	479.3; 317.2	35.0	0.0007 ± 0.0001	<0.0005
Bashkirskii	Delphinidin-3-glucoside	465.3; 303.2	25.0	0.0010 ± 0.0001	<0.0005
	Delphinidin-3-rhamnosyl-5-glucoside	627.3; 465.3; 303.2	17.5	0.0035 ± 0.0001	<0.0005
	Pelargonidin-3-glucoside	433.3; 271.1	37.5	0.0302 ± 0.0002	0.0012 ± 0.0001
	Petunidin-3-glucoside	479.3; 317.2	35.0	0.0025 ± 0.0001	<0.0005
Ognivo	Delphinidin-3-glucoside	465.3; 303.2	25.0	0.0003 ± 0.0001	<0.0005
	Pelargonidin-3-glucoside	433.3; 271.1	37.5	0.0030 ± 0.0001	<0.0005
Yubilar	Pelargonidin-3-glucoside	433.3; 271.1	37.5	0.0124 ± 0.0001	<0.0005
	Delphinidin-3-glucoside	465.3; 303.2	25.0	0.0003 ± 0.0001	<0.0005
Kuznechanka	Delphinidin-3-glucoside	465.3; 303.2	25.0	0.0077 ± 0.0001	<0.0005
	Delphinidin-3-rhamnosyl-5-glucoside	627.3; 465.3; 303.2	17.5	0.0100 ± 0.0001	<0.0005
	Pelargonidin-3-glucoside	433.3; 271.1	37.5	0.0852 ± 0.0003	0.0032 ± 0.0001
Sirenevyi tuman	Delphinidin-3-glucoside	465.3; 303.2	25.0	0.0030 ± 0.0001	<0.0005
	Malvidin-3-glucoside	493.3; 331.3	44.0	0.0120 ± 0.0001	<0.0005
	Pelargonidin-3-glucoside	433.3; 271.1	37.5	0.0060 ± 0.0001	<0.0005
Zhukovskii rannii	Pelargonidin-3-glucoside	433.3; 271.1	37.5	0.0352 ± 0.0002	<0.0005
Povin’	Delphinidin-3-glucoside	465.3; 303.2	25.0	0.0050 ± 0.0001	<0.0005
	Delphinidin-3-rhamnosyl-5-glucoside	627.3; 465.3; 303.2	17.5	0.0075 ± 0.0001	<0.0005
	Pelargonidin-3-glucoside	433.3; 271.1	37.5	0.0302 ± 0.0002	0.0009 ± 0.0001
	Petunidin-3-glucoside	479.3; 317.2	35.0	0.0050 ± 0.0001	<0.0005
	Cyanidin-3-rhamnosyl-5-glucoside	611.3; 499.3; 287.2	19.0	0.0110 ± 0.0001	<0.0005
Zhuravinka	Pelargonidin-3-glucoside	433.3; 271.1	37.5	0.0403 ± 0.0002	0.0021 ± 0.0001
Pri-15-7-16 Irbitskii × Avrora	Delphinidin-3-glucoside	465.3; 303.2	25.0	0.0020 ± 0.0001	<0.0005
	Pelargonidin-3-glucoside	433.3; 271.1	37.5	0.2041 ± 0.0003	<0.0005
	Malvidin-3-glucoside	493.3; 331.3	44.0	0.0108 ± 0.0001	<0.0005
	Petunidin-3-arabinoside	479.3; 317.2	16.0	0.0401 ± 0.0002	0.0014 ± 0.0001
	Cyanidin-3-glucoside	449.2; 287.2	27.5	0.0991 ± 0.0002	0.0023 ± 0.0001
Pri-15-41-8 Russkaya krasavitsa × Irbitskii	Pelargonidin-3-glucoside	433.3; 271.1	15.9	0.1301 ± 0.0004	<0.0005
	Malvidin-3-glucoside	493.3; 331.3	44.0	0.0052 ± 0.0001	<0.0005
	Delphinidin-3-glucoside	465.3; 303.2	15.9	0.0150 ± 0.0001	<0.0005
	Petunidin-3-arabinoside	479.3; 317.2	16.0	0.0167 ± 0.0001	<0.0005
	Cyanidin-3-glucoside	449.2; 287.2	10.8	0.0045 ± 0.0001	<0.0005
Pri-12-35-4 Debryansk × Mustang	Delphinidin-3-glucoside	465.3; 303.2	25.0	0.0035 ± 0.0001	0.0012 ± 0.0001
	Pelargonidin-3-glucoside	433.3; 271.1	37.5	0.1101 ± 0.0002	<0.0005
	Petunidin-3-arabinoside	479.3; 317.2	16.0	0.0078 ± 0.0001	<0.0005
Manifest	Delphinidin-3-glucoside	465.3; 303.2	25.0	0.0050 ± 0.0001	<0.0005
	Delphinidin-3-rhamnosyl-5-glucoside	627.3; 465.3; 303.2	17.5	0.0050 ± 0.0001	<0.0005
	Pelargonidin-3-glucoside	433.3; 271.1	37.5	0.0795 ± 0.0002	0.0226 ± 0.0001
	Petunidin-3-glucoside	479.3; 317.2	35.0	0.0010 ± 0.0001	<0.0005
Mayak	Delphinidin-3-glucoside	465.3; 303.2	25.0	0.0040 ± 0.0001	<0.0005
	Delphinidin-3-rhamnosyl-5-glucoside	627.3; 465.3; 303.2	17.5	0.0025 ± 0.0001	<0.0005
	Pelargonidin-3-glucoside	433.3; 271.1	37.5	0.1130 ± 0.0003	0.0029 ± 0.0001
	Petunidin-3-glucoside	479.3; 317.2	35.0	0.0120 ± 0.0001	<0.0005
Romanze	Pelargonidin-3-glucoside	433.3; 271.1	37.5	0.0621 ± 0.0002	0.0014 ± 0.0001
	Delphinidin-3-rhamnosyl-5-glucoside	627.3; 465.3; 303.2	17.5	0.0104 ± 0.0001	<0.0005
	Malvidin-3-glucoside	493.3; 331.3	44.0	0.0031 ± 0.0001	<0.0005
Mean				0.0738	0.0015
V, %				38.2	15.7
Purple and blue-purple tuber skin					
Tsyganka Lora	Petunidin-3-glucoside	479.3; 317.2	35.0	0.1140 ± 0.0003	0.0126 ± 0.0002
	Cyanidin-3-glucoside	449.2; 287.2	27.5	0.0301 ± 0.0002	<0.0005
Chernyi prints	Cyanidin-3-glucoside	449.2; 287.2	27.5	0.0708 ± 0.0002	0.0103 ± 0.0002
	Cyanidin-3-rhamnosyl-5-glucoside	611.3; 499.3; 287.2	19.0	0.0304 ± 0.0002	<0.0005
	Petunidin-3-glucoside	479.3; 317.2	35.0	0.1407 ± 0.0003	0.0241 ± 0.0003
Vasilek	Delphinidin-3-rhamnosyl-5-glucoside	627.3; 465.3; 303.2	17.5	0.0090 ± 0.0001	<0.0005
	Pelargonidin-3-glucoside	433.3; 271.1	37.5	0.0451 ± 0.0002	<0.0005
	Petunidin-3-glucoside	479.3; 317.2	35.0	0.1801 ± 0.0004	0.0261 ± 0.0003
	Cyanidin-3-rhamnosyl-5-glucoside	611.3; 499.3; 287.2	19.0	0.0020 ± 0.0001	<0.0005
Pri-15-12-14 Purple potato × Manifest	Petunidin-3-arabinoside	479.3; 317.2	16.0	0.3500 ± 0.0005	0.0911 ± 0.0003
	Cyanidin-3-glucoside	449.2; 287.2	27.5	0.1402 ± 0.0004	0.0398 ± 0.0002
	Pelargonidin-3-glucoside	433.3; 271.1	37.5	0.0050 ± 0.0001	<0.0005
	Delphinidin-3-glucoside	627.3; 465.3; 303.2	17.5	0.0631 ± 0.0001	0.0017 ± 0.0001
	Malvidin-3-glucoside	493.3; 331.3	22.5	0.0100 ± 0.0001	0.0010 ± 0.0001
Pri-14-52-2 Lomonosovskii × Purple potato	Petunidin-3-arabinoside	479.3; 317.2	16.0	0.3579 ± 0.0005	0.1430 ± 0.0004
	Cyanidin-3-glucoside	449.2; 287.2	27.5	0.0804 ± 0.0001	0.0812 ± 0.0003
	Pelargonidin-3-glucoside	433.3; 271.1	37.5	0.0841 ± 0.0002	0.0012 ± 0.0001
	Delphinidin-3-glucoside	627.3; 465.3; 303.2	17.5	0.0101 ± 0.0001	0.0015 ± 0.0001
	Malvidin-3-glucoside	493.3; 331.3	22.5	0.0021 ± 0.0001	0.0011 ± 0.0001
Fioletovyi	Delphinidin-3-glucoside	465.3; 303.2	25.0	0.0351 ± 0.0002	0.0026 ± 0.0001
	Malvidin-3-glucoside	493.3; 331.3	44.0	0.0603 ± 0.0002	0.0031 ± 0.0001
	Petunidin-3-glucoside	479.3; 317.2	35.0	0.0055 ± 0.0001	0.0021 ± 0.0001
	Cyanidin-3-glucoside	449.2; 287.2	27.5	0.1210 ± 0.0004	0.0352 ± 0.0002
	Cyanidin-3-rhamnosyl-5-glucoside	611.3; 499.3; 287.2	19.0	0.0089 ± 0.0001	0.0024 ± 0.0001
Mean				0.3349	0.0803
V, %				52.1	47.3

**Table 3:** Morphological characteristics of the studied potato specimens.

Variety/Hybrid	Origin	Color	
		Corolla	Tuber Flesh
Yellow and cream tuber skin			
Kazachok, st	Russia	White	Yellow
Yantar’, st	Russia	White	Bright yellow
Dachnyi, st	Russia	White	White
Sante, st	Netherlands	White	Yellow
Meteor	Russia	White	Yellow
Krepysh	Russia	Pale red-purple with white streaks	White
Sarma	Russia	White	Yellow
Azart	Russia	Pale blue-purple	White
Pamyati Rogacheva	Russia	Pale red-purple	Yellow
Nadezhda	Russia	Blue-purple with white streaks	Cream
Dubrava	Russia	Pale red-purple	Cream
Zol’skii	Russia	Pale red-purple with white streaks	Yellow
Krinitsa	Belarus	White	Yellow
Ragneda	Belarus	White	Yellow
Tamyr	Kazakhstan	White	Light yellow
Vitesse	Germany	White	Yellow
Shchedrik	Ukraine	White	White
Yellow tuber skin with pink spots, pink and red tuber skin			
Pamyati Kulakova	Russia	White	White
Ol’skii	Russia	White	Cream
Matushka	Russia	White	Cream
Bashkirskii	Russia	Pale red-purple	White
Ognivo	Russia	Red-purple with white streaks	Cream
Yubilyar	Russia	Red-purple	Yellow
Kuznechanka	Russia	Pale red-purple	White
Sirenevyi tuman	Russia	Pale red-purple with white streaks	Light yellow
Zhukovskii rannii	Russia	Red-purple	White
Zhuravinka	Russia	Red-purple	Yellow
Pri-15-7-16 Irbitskii × Avrora *	Russia	Red-purple with white streaks	Yellow
Pri-15-41-8 Russkaya krasavitsa × Irbitskii *	Russia	Red-purple with white streaks	White
Pri-12-35-4 Derbyansk × Mustang *	Russia	Blue-purple	Yellow
Manifest	Russia	Red-purple with white streaks	Cream
Mayak	Russia	Pale red-purple	Cream
Povin’	Ukraine	Red-purple	Yellow
Romanze	Germany	Red-purple	Yellow
Purple and blue-purple tuber skin			
Tsyganka Lora	Russia (folk variety)	Red-purple	Cream
Chernyi prints	Russia (folk variety)	Red-purple with white streaks	Cream
Vasilek	Russia	Blue-purple with white streaks	Cream
Pri-15-12-14 Purple potato × Manifest *	Russia	Red-blue-purple	Yellow with purple pigmentation
Pri-14-52-2 Lomonosovskii × Purple potato *	Russia	Pale red-purple	Yellow with purple pigmentation
Fioletovyi *	Russia	Blue-purple with white streaks	Purple

Note: *—hybrid created by the Federal Scientific Center of Agricultural Biotechnology of the Far East named after A.K. Chaiki, Ussuriysk, Russia.

## Data Availability

All the datasets are provided within the manuscript.
